# Psychological Differences toward Pedestrian Red Light Crossing between University Students and Their Peers

**DOI:** 10.1371/journal.pone.0148000

**Published:** 2016-01-29

**Authors:** Qinghui Suo, Daming Zhang

**Affiliations:** School of Engineering and Technology, Southwest University, Chongqing 400715, China; Beihang University, CHINA

## Abstract

Based on our site investigation conducted in 2013, we found that the pedestrian red light crossing at the midblock connecting the campus of Southwest University and living area was low, where most of pedestrians are university students and staff. This paper reports a supplementary work applying the Theory of Planned Behaviour (TPB) to identify any psychological differences toward pedestrian red light crossing between university students and their peers. Three social groups participated in the investigation. The first group is the university students in Grade one (Group 1), the other two groups are their previous senior middle school classmates who are now working full time (Group 2) or who are now out of work and school (Group 3). The statistical results indicated The TPB components accounted for 42.9%, 55.3% and 55.4% of the variance of red signal crossing intention for Group 1, Group 2 and Group 3 in the depicted road crossing scenario. The data also showed that there are obvious differences among the participants’ responses to “refrain from crossing” between university students and others, and the subsequent regression analysis revealed the ability to “refrain from crossing” played the most important role in the intention of red light crossing in the depicted scenario.

## Introduction

Globally, there is growing concern regarding the high rate of pedestrian fatalities in traffic accidents. Pedestrians constitute 22% of all road traffic fatalities, and in certain countries, this proportion is as high as two thirds of all road traffic deaths [1]. While crossing the road, pedestrians should coordinate between the oncoming vehicles and their walking. Signalized pedestrian crossing prevents pedestrians and vehicles from simultaneously crossing, and it is illegal to cross the road while the pedestrian signal is red. Low level of compliance to traffic rules and the unsafe attitudes both among drivers and pedestrians are among the reasons for the low level of pedestrian safety [2].

The pedestrian injury event can result from both human behaviours and environment [3] and pedestrian behaviours are likely to be influenced by individual properties [4]; different types of pedestrians, such as careful versus aggressive and complying with traffic rules versus non-complying with rules, influence the traffic condition on different levels [5, 6]. A clearer understanding of the motivational and attitudinal determinants of different social group’s risky choices may facilitate the development of effective road safety interventions [7]. There is evidence to suggest that specific social groups have different approaches to law abidance due to circumstances of conflict [8], and different individuals have different propensity for risk taking; many researchers have investigated behaviour and psychology of pedestrian red light crossing. For example, Rosenbloom [9] found that highly sensation-seeking pedestrians crossed at red lights more than low sensation-seekers; Ultra-Orthodox pedestrians committed more violations than secular pedestrians in Israel [10]; people who showed greater tendencies towards social conformity had stronger road crossing intentions than low conformity people [11]; Hamed [12] indicated that individuals who have a driver’s license are more likely to take risks as pedestrians than those that do not drive, whereas Taubman-Ben-Ari and Shay [13] reached a conflicting conclusion; Rosenbloom [14] revealed that soldiers demonstrated road-crossing behaviour that was significantly safer than that of civilians. It was also found that male pedestrians have a higher percentage of violations than female pedestrians[15–20] and young adults and adolescent pedestrians are more likely to commit violations than older pedestrians[21, 22]. These research findings revealed that different types of road-use behaviour are distinct from each other to some extent.

## Background

The authors are affiliated with Southwest University, which is located in the Beibei District of China, approximately 45 km from the Chongqing metropolitan area. According to the first-author’s long-term observations, the probability of illegal crossing at the mid-block facing the main entrance of campus is relatively low, and the pedestrians’ road performance at this mid-block is much safer than that of the normal public. In order to quantify the pedestrians’ traffic rule violation rate at this mid-block, a site investigation was conducted in April 2013. A total of 1,621 pedestrian crossing behaviours were recorded in the investigation. The percentage of illegal crossing at the survey point is 7.2%, which is less than the results reported by other investigations conducted in China [18, 23, 24]. In this article, we attempted to identify any psychological differences toward pedestrian red light crossing between university students and their peers. Firstly, a questionnaire was constructed based on the theory of planned behaviour (TPB); next, a survey was distributed among three different social groups (university students and their peers), and finally, statistical analysis on the data obtained was conducted to fulfil the research purpose.

## Methods

In addition to using the macro and micro models to simulate the pedestrians’ behaviour [25], social psychological models could be applied to investigate pedestrians’ behaviour and attitude. One way for investigating human behaviour decision making is through the use of the Theory of Planned Behaviour (TPB) [26], which is one of the most widely cited and applied social psychological models of health and safety related behaviour [27]; the theory has been successfully applied to the prediction of a wide range of health-related behaviours and a number of studies have supported the validity of the TPB [28, 29]; Scholars have implemented the theory to investigate pedestrians’ road crossing behaviour [7, 11, 21, 30]. For example, Evan and Norman applied the TPB to the prediction of pedestrians’ road crossing intention in the scenarios of three potentially dangerous road crossing [7]; pedestrians’ attitudes towards traffic violations and self-rating of violations, errors and lapses were measured based on the TPB by Díaz [21]; Zhou et al. also used the TPB to evaluate the effects of age, gender and conformity tendency on Chinese pedestrians’ intention to cross the road in potentially dangerous situations[11]; Xu et al. predicted the intention of pedestrian’ illegal road-crossing with an extended version of TPB [30].

The questionnaire survey was carried out in May and June 2015. In this survey, we formulated a questionnaire (in S1 Appendix) based on the TPB and the participants in the survey were selected from three social groups. Firstly, we contacted university students in grade one (finished senior middle school in July 2014, Group 1), who have been enrolled at the university for the 9 months while the investigations were conducted. University students have the following common characteristics:

1. most are between 17 and 25 years old,
2. they passed the National Higher Education Entrance Examination of P.R. China and obtained a comparatively high overall mark, which suggests most worked hard and had a good attitude about learning during middle school,
3. most share the accommodations provided by the university and study in the same classroom with their classmates from all over China, so they can inevitably influence each other in terms of behaviour and modes of thinking, and
4. they are an intellectually active group in society.

The university students were informed of the purpose of the study and were asked to complete the questionnaire, we also asked if she/he can request two of her/his former senior middle school classmates to complete the same survey form; one of the former classmates has been working full time for at least 4 months (Group 2), and the other one has been out of work and school since graduation from senior middle school (Group 3). 300 (3×100) questionnaires were handed out and 228 (3×76) valid results were reclaimed. The study was conducted with permission of Academic Council of Southwest University, which is responsible for academic ethnics and Integrity. Before the questionnaire survey, all the participants were informed of the purpose of the study; they participated in the survey voluntarily; they were ensured that their answers would remain anonymous and that the data would only be used for scientific purposes. Except the gender and age, no personal information was recorded.

The contents of the questionnaire applied in this work were constructed based on the previous studies [7, 11, 21, 30]. For the questionnaire, we depicted one common scenario of a pedestrian’s red light crossing: “While you reach the zebra crossing with a pedestrian traffic signal and find a “red man” signal. About ten pedestrians are waiting for the “green man” signal, and two or three pedestrians are crossing the crosswalk against the signal. You cross following the pedestrians during a gap in the traffic. How do you rate the above to your usual behaviour?” 18 questions were included in the questionnaire, and they were asked to rate on a five-point scale. The pedestrian road crossing behaviour in China must be mentioned here. In Chinese cities, many pedestrians cross a road not based on the traffic signals but by whether there are a sufficient number of pedestrians who are violating the rules. When the leading pedestrians step off the curb and enter the road, others normally follow without estimating the risk or underestimating the risk. This phenomenon is the so-called Chinese Style Road Crossing, and it has been reported and criticized in the Chinese mainstream media in recent years. This phenomenon provides the support for the Theory of Social Psychology: A group's norms, rules and moral standards justify the actions of its individual members, particularly when these actions conflict with some established social norms [31], and each group member feels that the responsibility for violating the norm (e.g., traffic rules) is shared with the rest of the members [32].

### Attitude

According to the TPB, human behaviour is guided by three types of considerations: behavioural beliefs, normative beliefs and control beliefs. Behavioural belief consider about the likely consequence of the behaviour which produce a favourable or unfavourable attitude toward the behaviour. For the behavioural beliefs, three items including one positive and two negative beliefs were investigated: “I _______ that the behaviour would get me to my destination more quickly, injury myself and annoy drivers?” “Get me to my destination more quickly” is a positive belief for the behaviour, and the other two items, “injury myself” and “annoy drivers”, are negative. The participants were asked to rate the response using a five point Likert scale from “strongly agree (2 for positive belief and -2 for negative belief)” to “strongly disagree (-2 for positive belief and 2 for negative belief).

For each belief, there was a corresponding outcome evaluation item. For example, “Get me to my destination more quickly is _______ .” The participants rated their response on a 5-point scale from 1 (extremely good) to 5 (extremely bad). The measure of each attitude belief was calculated by multiplying the belief with the corresponding outcome evaluation.

### Subjective Norm

Normative beliefs are about the normative expectations of others which results in perceived social pressure or subjective norm. Four referents, including the police, parents, drivers and other pedestrians, were used to measure the normative belief. The participants were asked to rate the likelihood that referents would approve or disapprove of their red-light-crossing in the depicted scenario using the five point Likert scale, scored 2 (strongly approve) to -2 (strongly disapprove). The participants were also asked to express the extent to which she/he will comply with referents’ wishes using the five point Likert scale, scored 1 (extremely likely) to 5 (extremely unlikely). The average value of each item was calculated by multiplying the normative belief by the motivation to comply.

### Perceived Behavioural Control

Perceived behaviour control is about factors that may facilitate or impede performance of behaviour. For perceived behavioural control, two control beliefs were used (one positive, one negative). One is: “In the depicted scenario, it is _____ for me to cross the road”, and the participants rated their responses on a 5 point Likert scale from 2 (extremely easy) to -2 (extremely difficult). The other is “In the depicted scenario, it is _____ for me to refrain from crossing the road.” The response is scored -2 (extremely easy) to 2 (extremely difficult).

### Past Behaviour

Past behaviour was measured by one item: “The frequency of my crossing in the depicted scenario was ______ in the last 3 months.” The participant responses were on the 5-point Likert scale from -2 (extremely high) to 2 (extremely low).

### Behavioural Intention

In combination, attitude toward the behaviour, subjective norm and perception of behavioural control lead to the formation of a behavioural intention. As a general rule, the more favourable the attitude and subjective norm, and the greater the perceived control, the stronger should be the person’s intention to perform the behaviour. Behavioural intention was measured by one item: “I think that I would _____ cross the road in the depicted scenario in the future.” The participant responses were on the 5-point Likert scale from -2 (always) to 2 (never).

## Results and Discussion

### Means and Standard Deviations

The means and standard deviations of each component of TPB are shown in Table 1. The means of the TPB components indicated that all three groups have negative attitudes toward the behaviour, and the group of university students exhibits the strongest negative response (M = -1.56) compared to Group 2 (M = - 0.50) and Group 3 (M = - 0.45). They also demonstrated negative subjective norms to the behaviour, and there is a moderate difference among the means of the three groups, which is not as obvious as attitude. These negative scales showed that this type of behaviour would attract social disapproval, and the participants thought they would face social pressure if they cross in the depicted manner. Participants’ responses to perceived behaviour control are also slightly below the midpoint (M = -0.50, M = -0.15 and M = -0.09). By comparing the means, group 2 and group 3 are more likely to cross in the depicted scenario than group 1.

**Table 1:** Means and SD of components of the TPB.

	Group 1		Group 2		Group 3	
	Mean	SD	Mean	SD	Mean	SD
**Attitude**	-1.56	6.91	-0.50	4.96	-0.45	4.35
**Subjective Norm**	-1.17	3.01	-0.88	2.66	-0.71	3.20
**Perceived Behavioural control**	-0.50	1.48	-0.15	1.23	-0.09	1.24
**Past Behaviour**	1.09	0.94	0.38	1.14	0.45	1.23
**Behavioural Intention**	1.17	0.91	0.53	1.17	0.38	1.32

Although the TPB components are negative, the scales of the past behaviours and behavioural intentions of the three groups are all positive (1.09, 0.38 and 0.45; 1.17, 0.53 and 0.38), and group 1 has the highest scale in terms of past behaviour (M = 1.09), which indicates they behaved better than the other two groups, which is consistent with our field investigation conducted in 2013.

The means and standards deviations of each item of the TPB are shown in Table 2. By comparing the means of each item, there are obvious differences among participant responses to several items. For example, the university students’ response to “Q1: Get me to my destination more quickly” is -3.46, whereas the scales of group 2 and 3 are -0.55 and -0.63, respectively. Although saving dozens of seconds accompanied with the risk of a road accident is actually meaningless for pedestrians to cross during the “red man”, it looks like group 2 and 3 overestimated the benefit brought by crossing during the red light, whereas university students gave a more rational evaluation toward the behaviour.

**Table 2:** Means and SD of items of the TPB.

TPB Components	TPB Items	Group 1		Group 2		Group 3	
		Mean	SD	Mean	SD	Mean	SD
**Attitude**	Q1. More quickly	-3.46	5.29	-0.55	3.85	-0.63	3.26
	Q2. Injury myself	0.39	7.49	0.24	5.95	0.45	4.50
	Q3. Annoy driver	-1.61	7.26	-1.17	4.84	-1.17	5.01
**Subjective Norm**	Q4. Parents	0.38	3.03	-0.66	2.54	0.08	3.09
	Q5. Traffic police	-1.75	3.22	-1.47	2.83	-1.41	3.59
	Q6. Drivers	-1.58	3.14	-1.04	2.71	-1.07	3.35
	Q7. Other pedestrians	-0.96	2.44	-0.34	2.47	-0.45	2.53
**Perceived Behavioural control**	Q8. Crossing	0.26	1.57	0.28	1.25	0.38	1.14
	Q9. Refrain from crossing	-1.26	0.87	-0.58	1.05	-0.55	1.17
	Q10. Past Behaviour	1.09	0.94	0.38	1.14	0.40	1.23
	Q11. Behavioural Intention	1.19	0.91	0.53	1.17	0.38	1.32

The university students’ scale to the “Q9: Refrain from crossing” is -1.26, whereas the scales of group 2 and 3 are -0.58 and -0.55, which probably indicates that university students have stronger self-control ability toward the red light crossing than do the other two groups.

### Correlations of Road Crossing Intentions

The correlation coefficients between behavioural intention and the TPB components were calculated for each group (See Table 3). For each group, the intention to cross in the depicted scenario was significantly correlated with attitude, subjective norm and perceived behaviour control. Perceived behaviour control showed the strongest correlation with behavioural intention for Group 1 and Group 3, whereas subjective norm showed the strongest correlation for Group 2. The correlations of each item with the intention are shown in Table 4. As observed, the correlation between the items of “Q9. Refrain from crossing” and the behavioural intention is significant for each group.

**Table 3:** Zero-order correlation coefficients between TPB components and behavioural intentions.

	Attitude	Subjective Norm	Perceived Behavioural control
**Group 1**	0.356	0.249	0.631
**Group 2**	0.228	0.563	0.483
**Group 3**	0.487	0.405	0.491

**Table 4:** Zero-order correlation coefficients between TPB items and behavioural intentions.

	Q1	Q2	Q3	Q4	Q5	Q6	Q7	Q8	Q9
**Group 1**	-.352	-.139	-.141	-.113	.074	-.114	-.184	-.018	-.625
**Group 2**	.074	.108	-.118	-.204	.189	-.279	-.379	-.228	-.465
**Group 3**	-.465	.005	-.032	-.395	-.040	-.082	-.056	-.080	-.467

### Regression Analysis

In the hierarchical regression analysis, three blocks of variables, including attitude, subjective norm and perceived behavioural control, were used to predict behavioural intention, and the prediction models were statistically significant. The TPB components accounted for 42.9%, 55.3% and 55.4% of the variance of behavioural intention for Group 1, Group 2 and Group 3, respectively.

Nine TPB items were used in a further stepwise multiple regression analysis to predict the behavioural intention (see Table 5). For Group 3, the final prediction model contained three predictors (Q9, Q1 and Q4) and was reached in three steps with six variables removed. The model was statistically significant (*F* (3, 72) = 24.677, *p* < 0.001), and accounted for 50.7% of the variance of intention (*R*^2^ = 0.504). “Q9. Refrain from crossing” received the strongest weight in the model, followed by “Q1. More quickly” and “Q4. Parents”. For group 2, the data shows that “Q9. Refrain from crossing” accounted for 21.6% of the variance in behavioural intention. In the fourth and final regression, the addition of traffic police, drivers and other pedestrians led to an increase of 27.8% in the amount of variance explained (F (4, 71) = 17.335, p < 0.001). For the university students (Group 1), only item 9 was included in the final regression model, and it accounted for 39.0% of the variance of behavioural intention (F (1, 74) = 47.319, P < 0.001).

**Table 5:** Stepwise Multiple Regression Analysis.

	Variables included	R^2^	
**Group 3**	Q9	0.218	
	Q9 and Q1	0.346	
	Q9, Q1 and Q4	0.507	F (3, 72) = 24.677, p < 0.001
**Group 2**	Q9	0.216	
	Q9 and Q7	0.362	
	Q9, Q7 and Q6	0.421	
	Q9, Q7, Q6 and Q5	0.494	F (4, 71) = 17.335, p < 0.001
**Group 1**	Q9	0.390	F (1, 74) = 47.319, P < 0.001

## Discussion

Pedestrian red light crossing behaviour and psychology have been thoroughly analysed by previous researchers [7, 11, 21, 30], and helpful findings have been revealed. Most of our results obtained were consistent with those previous results. We are additionally interested in finding any differences among university students and their peers. For the self-reported results, we observe the followings:

The average scale of past behaviour for university students is 1.09, which is larger than that of the other two groups (0.38 and 0.40); university students made fewer illegal crossings compared to the other two groups according to the self-reported data about past behaviour. This result is consistent with our field investigation conducted in 2013.

By comparing the means of the TPB item of “more quickly”, there is an obvious difference between the university group and the other two groups; however, the effect of this item in the final stepwise regression model is not obvious.

The stepwise regression analysis showed that the ability to “refrain from crossing” played the most important role in red light crossing in the depicted scenario, and there is an obvious difference in response to “refrain from crossing” between university and other groups (see Table 2). It looks like that the university students have stronger self-control ability toward the behaviour. Self-control is the ability to control one's emotions, behaviour, and desires in the face of external demands to function in society (https://en.wikipedia.org/wiki/Self-control).

We speculate there are two possible reasons; one is that the young people who have stronger self-control ability have a greater probability of passing the National Higher Education Entrance Examination of P.R. China and thus entering a university. Therefore, university students have stronger self-control ability than their peers. However, in our questionnaire survey conducted in 2014, 199 of 523 university students (38%) claimed that they attempted fewer illegal pedestrian crossings than before entering university, and only 57 students (11%) reported a worse trend; the students did behave better after entering the university based on our investigation, so their experience in university improved their behaviour. This hypothesis does not successfully establish.

Site investigations revealed that both soldiers and military officers committed less traffic violations than civilians and they obey the traffic rules at a higher rate than did civilians [15]. Rosenbloom [15] explained the behaviour by the principles of Role Theory, military staff was identified by the uniforms and so their behaviours fulfil the duties, expectation, rights and norms of their social role which results in a higher compliance rate to the (traffic) rules. In our investigations, there were not obvious signs, such as school uniforms, to distinguish research populations from common pedestrians, so we think the Role Theory does not apply here. Xu et al. revealed that a pedestrian’s intention to violate traffic rules is driven more by the frequency of past behaviour (habit), and less by controlled analytic process [30]. We think that the second possible reason is that university students have developed the habit of compliance to the social rules after entering the university. In the American Journal of Psychology, a habit is defined as a more or less fixed way of thinking, willing, or feeling acquired through previous repetition of a mental experience [33]. Habit formation is the process by which behaviour becomes automatic or habitual through regular repetition. This is modelled as an increase in automaticity with the number of repetitions up to an asymptote [34]. Lally et al. found the average time for participants to reach the asymptote of automaticity was 66 days, with a range of 18–254 days [34]. In Chinese universities, students are used to studying in the classroom, attending social activities and even looking for accommodations under the arrangements of the university, while in senior middle school, they normally focused more on examination preparations and cared more about examination results rather than other social activities. We think the experience under the unified arrangements of the university has an impact on the formation of the students’ habit of compliance with the rules. High levels of traffic safety are accompanied with the establishment of good habits of the majority of university members. Individuals intending to violate the rule would incur criticism and pressure from surroundings, and thus they tend to stick to traffic rules [32].

## Conclusions

The psychological differences toward pedestrian red light crossing between university students and their peers were examined using a survey based on the TPB. Three groups of research populations, including university students and their peers, participated in the survey. The results showed that TPB components explained 42.9%–55.4% of the variance of behavioural intention and attitude, subjective norm and perceived behaviour control can be taken as predictors for the behaviour. The statistical analysis also revealed that the university students behaved better than the other two groups towards pedestrian red light crossing, which is consistent with our field investigation conducted in 2013. Most importantly, the statistical data indicated that there are obvious differences among the research populations’ responses to the TPB item of “refrain from crossing”; the subsequent regression analysis confirmed that “refrain from crossing” played the most important role in the behavioural intention. It looks like that the university students obey traffic laws at a higher rate than their peers and we speculate that the experience under the unified arrangements of the university has an impact on the formulation of the students’ habit of compliance with the traffic rules.

This study has several limitations. The participants are selected from the students of Southwest University and their former senior middle school classmates. The number of valid responses was only 228 (3 × 76) for the investigation, which means that the sample size is small. The investigation was conducted in China, a developing country with poor traffic safety culture; we do not know whether consistent results could be obtained in other countries, especially in countries with better traffic order and traffic safety culture. The statistical results do show that university students have stronger self-control ability toward red light crossing, although we cannot explain this result irrefutably.

## Supporting Information

S1 Appendix (DOCX)
