# Supplementary material for: Psychological Differences toward Pedestrian Red Light Crossing between University Students and Their Peers
Source: PLoS One. 2016 Jan 29;11(1):e0148000. doi: 10.1371/journal.pone.0148000 (PMC4732691; doi:10.1371/journal.pone.0148000)
Supplement: S1 Appendix — (DOCX) [file pone.0148000.s001.docx]

**S1 Appendix**

While you reach the zebra crossing with pedestrian’ traffic signal and find “Red Man”, about ten pedestrians are waiting for “Green Man” and two or three pedestrians are crossing crosswalk against the signal. You straight cross following the pedestrian during the gap in the traffic.

1. I that this behaviour would get me to the destination more quickly.

strongly agree 2 : 1 : 0 : -1 : -2 strongly disagree

“Get me to my destination more quickly and save my time” is .

extremely good 1 : 2 : 3 : 4 : 5 extremely bad

1. I that this behaviour would get me injured.

strongly agree -2 : -1 : 0 : 1 : 2 strongly disagree

“Get me injured” is .

extremely good 1 : 2 : 3 : 4 : 5 extremely bad

1. I that this behaviour would annoy drivers.

strongly agree -2 : -1 : 0 : 1 : 2 strongly disagree

“Annoy drivers” is .

extremely good 1 : 2 : 3 : 4 : 5 extremely bad

1. My parents would this kind of behaviour.

strongly approve 2 : 1 : 0 : -1 : -2 strongly disapprove

My motivation to comply with my parents’ wish to this kind of behaviour is .

extremely likely 1 : 2 : 3 : 4 : 5 extremely unlikely

1. Traffic police would this kind of behaviour.

strongly approve 2 : 1 : 0 : -1 : -2 strongly disapprove

My motivation to comply with traffic police’ wish to this kind of behaviour is .

extremely likely 1 : 2 : 3 : 4 : 5 extremely unlikely

1. Drivers would this kind of behaviour.

strongly approve 2 : 1 : 0 : -1 : -2 strongly disapprove

My motivation to comply with drivers’ wish to this kind of behaviour is .

extremely likely 1 : 2 : 3 : 4 : 5 extremely unlikely

1. The other pedestrians would this kind of behaviour.

strongly approve 2 : 1 : 0 : -1 : -2 strongly disapprove

My motivation to comply with other pedestrians’ wish to this kind of behaviour is .

extremely likely 1 : 2 : 3 : 4 : 5 extremely unlikely

1. In the depicted scenario, it is for me to cross the road.

Extremely easy 2 : 1 : 0 : -1 : -2 extremely difficult

1. In the depicted scenario, it is for me to refrain from crossing the road.

Extremely easy -2 : -1 : 0 : 1 : 2 extremely difficult

1. The frequency of my red light crossing in the depicted scenario was in the last 3 months.

Extremely high -2 : -1 : 0 : 1 : 2 extremely low

1. How likely I would cross the road in the manner depicted in the scenario in the future?

Extremely high -2 : -1 : 0 : 1 : 2 extremely low
